# Governance models for nature-based solutions: Seventeen cases from Germany

**DOI:** 10.1007/s13280-020-01412-x

**Published:** 2020-12-31

**Authors:** Aude Zingraff-Hamed, Frank Hüesker, Christian Albert, Mario Brillinger, Joshua Huang, Gerd Lupp, Sebastian Scheuer, Mareen Schlätel, Barbara Schröter

**Affiliations:** 1 Technical University of Munich, Chair for Strategic Landscape Planning and Management, Emil-Ramann-Str. 6, 85354 Freising, Germany; 2 Helmholtz Centre for Environmental Research, Permoserstr. 15, 04318 Leipzig, Germany; 3 Ruhr-Universität Bochum, Institute of Geography, Universitätsstr. 150, 44805 Bochum, Germany; 4 Humboldt-University Berlin, 10099 Berlin, Germany; 5 Leibniz Centre for Agricultural Landscape Research, Working Group “Governance of Ecosystem Services”, Eberswalder Str. 84, 15374 Müncheberg, Germany

**Keywords:** Financing instruments, Flood risk mitigation, Institutional structures, Polycentric governance, River Management, Stakeholder participation

## Abstract

Nature-based solutions (NBS) for mitigating climate change are gaining popularity. The number of NBS is increasing, but research gaps still exist at the governance level. The objectives of this paper are (i) to give an overview of the implemented NBS for flood risk management and mitigation in Germany, (ii) to identify governance models that are applied, and (iii) to explore the differences between these models. The results of a hierarchical clustering procedure and a qualitative analysis show that while no one-size-fits-all governance model exists, polycentricism is an important commonality between the projects. The study concludes by highlighting the need for further research on traditional governance model reconversion and paradigm changes. We expect the findings to identify what has worked in the past, as well as what is important for the implementation of NBS for flood risk management in future projects.

**Electronic supplementary material:**

The online version of this article (10.1007/s13280-020-01412-x) contains supplementary material, which is available to authorized users.

## Introduction

Change in climate patterns cause the increase of extreme hydro-meteorological events which results in more floods and droughts (Beniston 2007; De Paola et al. 2018; EC 2020). While flooding is a natural process that is essential for biological health and riverine functions (Junk et al. 1989), it also represents one of the most common natural hazards that lead to catastrophes in Europe (EEA 2016). Floods have caused not only damages and disruptions, but also various health effects including deaths, injuries, poor sanitation, and poor water quality (Hajat et al. 2005; Doocy et al. 2013). Hydro-meteorological experts estimate that climate change may induce more flood risk due to an increase in the intensity and frequency of extreme weather events (EEA 2016). Annual monetary damages from flooding in Europe is expected to rise from 6 billion to about 108 billion USD by 2080 if no further prevention and adaptation measures are implemented (EC 2014).

To address growing flooding risk and related impacts, nature-based solutions (NBS) are becoming more popular as an effective complement or partially replacement of conventional technical approaches such as static flood protection infrastructures (UN 2018). The benefits of intact ecosystems is since early twenty-first century recognized (Cohen-Shacham et al. 2016) and some concepts such as “more room for the river” in France, the Netherlands, and Germany acknowledged the benefits of dynamic environmental processes. However, in the past two decades, the implementation of ecosystem-based management has become more popular worldwide, and the need for consistent terminology has resulted in the use of the term NBS. NBS can consist of different levels of natural components (Eggermont et al. 2015). For flood risk mitigation, examples of NBS include providing more space for rivers, e.g., Nesttunvassdraget in Norway (CoB 2007), revitalizing floodplains, e.g., Grand Park Garonne in France (Van de Kreek and Etienne 2012) establishing green infrastructure in cities, e.g., The Green Ring, Antwerpen in Belgium (Haine 2014), and implementing decentralized rainwater management, e.g., Rewitalizacja rzeki Białej in Poland (Sadowska-Dubicka 2015). NBS are defined as “actions which are inspired by, supported by or copied from nature” (EC 2015b), or more specifically, actions that (i) alleviate a well-defined societal challenge, (ii) utilize ecosystem processes, and (iii) are embedded within viable governance models (Albert et al. 2019). Governance models are ideal governance types explaining the interrelation of different actors and institutions in the context of rules and rule-making systems to coordinate interdependencies and hierarchical market and community management (Wamsler et al. 2017).

The concept of NBS has recently gained attention in science and public policy (Nesshöver et al. 2017; Frantzeskaki et al. 2019) following its introduction by the International Union for Conservation of Nature (Cohen-Shacham et al. 2016, 2019) and the European Union (EU). A large number of long-term research projects have recently been funded (EC 2015a, b), such as Physicos^1^ and ReConect^2^. A common strategy of those projects is to stimulate transdisciplinary research and to optimize and upscale pilot solutions to other sites while financially supporting implementation and providing governance support to enhance collaborative planning. Increased efforts have been undertaken recently to document and synthesize cases of NBS application in online databases (e.g., Oppla^3^). They aim to cross-fertilize and are useful for extracting technical and societal knowledge from success stories and cases that are recognized as good practice.

Unfortunately, the number of NBS is still low and implementations are often slowed down by barriers in governance (Kabisch et al. 2016; Ershad Sarabi et al. 2019). This indicates that investigating governance models may be a key to learning about more effective NBS implementation. Presently, there is little comparative research on NBS governance. Furthermore, because of different policy frameworks and local societal challenges, comparison and upscaling of research results are very limited (exception Martin 2019). Study showed that different water governance culture exist between the EU countries and that while EU directives highly influence the EU member policy, its incorporation in national law and its implementation vary between the countries and cause bias in regional governance comparison (Zingraff-Hamed et al. 2017b). Governance models have been mostly investigated in theoretical terms (Kooiman 2003; Treib et al. 2007), in the context of environmental policy (Arnouts et al. 2012), governance of ecosystem services (Vatn 2010; Schröter et al. 2019), and water governance (Pahl-Wostl 2015, 2019), but not in the context of NBS and not in a systematic way.

Consequently, our research question is as follows: Which governance models led to NBS implementation for flood risk management and mitigation? Specifically, our objectives are (i) to give an overview of the implemented NBS for mitigating flood risk in Germany, focusing on their governance models, (ii) to identify governance models that are applied in implemented cases, and (iii) to explore the differences between the models that are applied, in order to discuss future water governance challenges and to formulate recommendations for further implementation of NBS. In order to investigate the implementation of NBS in more detail from a governance perspective while avoiding comparison bias caused by policy variability, we decided to conduct an analysis that focused on Germany. As a federal state, Germany is characterized by a hierarchical share of competencies and state governments of the 16 states are responsible for policy implementation (Jänicke et al. 2001; Schroeter 2018). The state governments have much flexibility in the NBS planning process making Germany an interesting field for investigation of the design and implementation of NBS under different regional governance models (e.g., Newig et al. 2016).The results from our analysis are expected to give insights for implementing NBS in Germany and all around the globe.

## Materials and methods

**Fig. 1:**
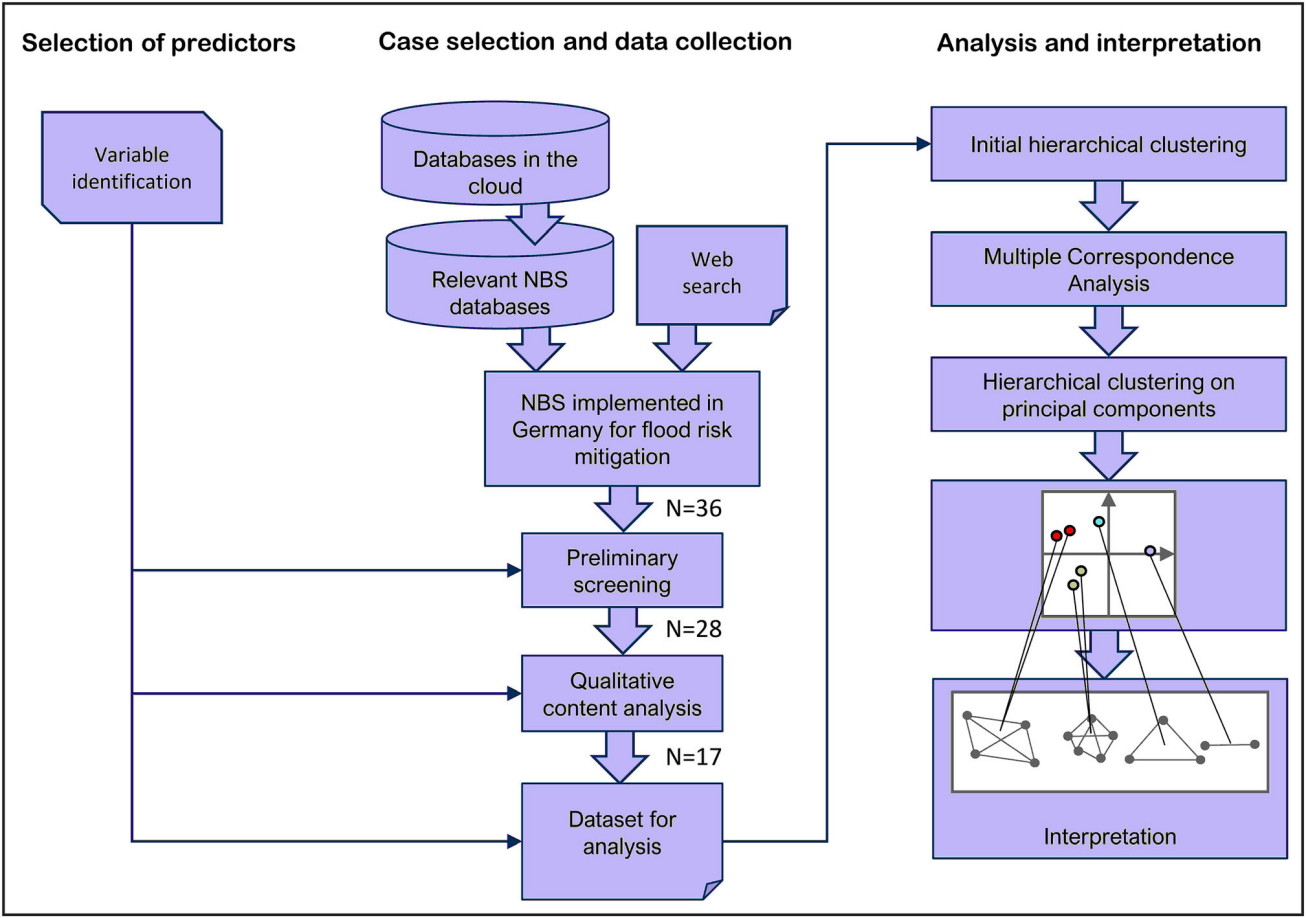
Methodology outlining the analytical framework of the case study

The methodology that we used in our analytical framework consists of the identification of predictor variables for the identification of key governance features, case selection and data collection, and the subsequent analysis of cases related to key governance features and their interpretation (Fig. 1).

### Selection of predictors

To identify relevant variables for the governance analysis, we first identified and reviewed relevant literature. A preliminary list of variables was discussed during a workshop in February 2019 with seven NBS governance researchers from PlanSmart, Rivercare, Phusicos and ReConect. This result of this workshop was a list of twelve governance features, from which six were specifically selected (framing and implementing organizational structures, project coordination, participation level, institutional setting, financing model, and property rights constellation) because of their importance and potential for further analysis. In a second workshop, the working group further detailed the six selected governance features and categorized them by a number of possible predictor variables. For each variable, a characteristic question was formulated to simplify the subsequent data collection. For most variables, we defined a selection of qualitative and quantitative list of modalities (Table S1).

### Case selection and data collection

**Fig. 2:**
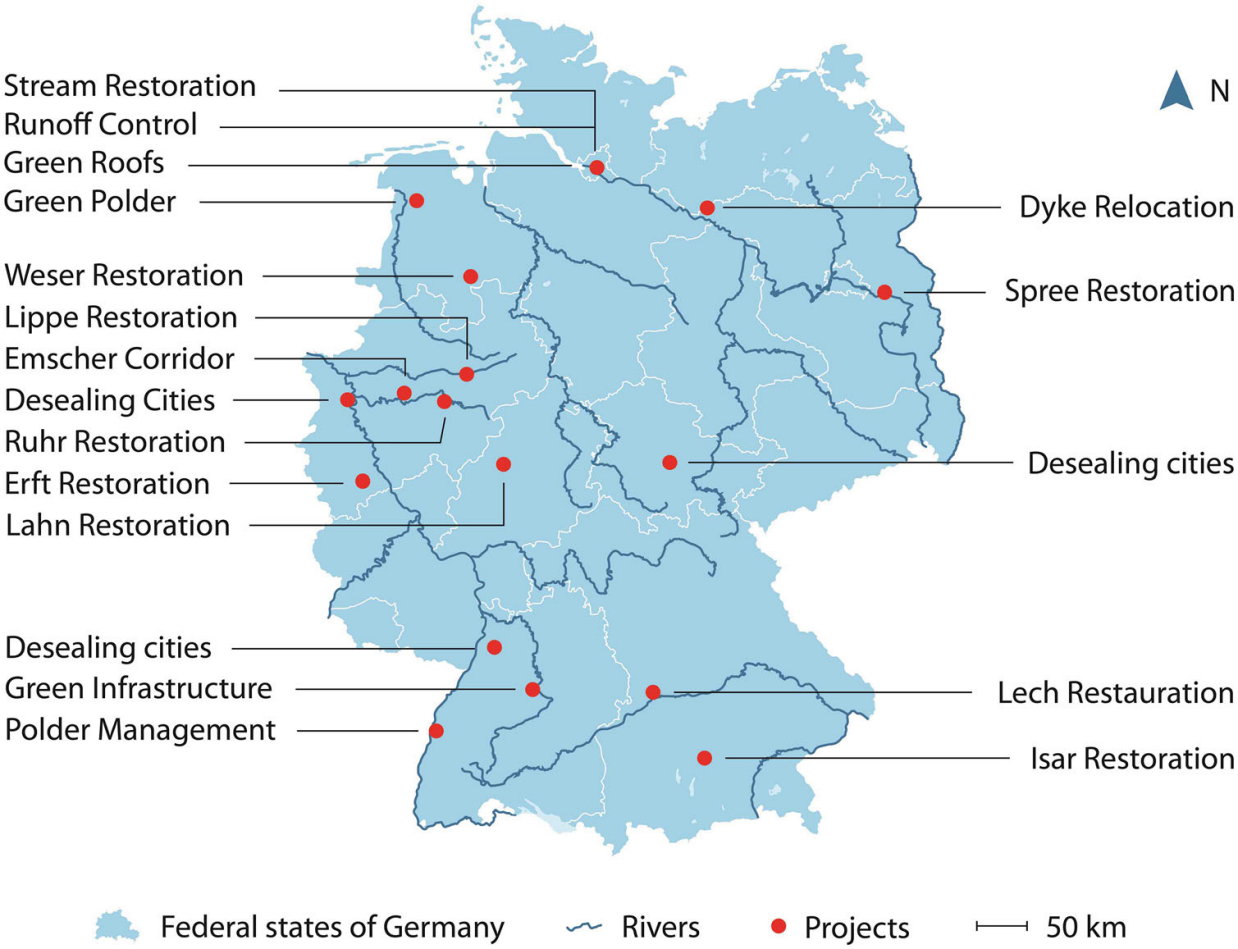
Location of the selected case sites

We identified successfully implemented NBS for flood risk mitigation in Germany by querying existing NBS databases developed by several EU funded research projects in order to document the best practices of NBS implementation. An online search (June 2019, terms applied: “Nature-based solutions” AND “database”) identified 59 relevant NBS databases. Then, they were filtered for German NBS for flood mitigation. We added to the selection German cases of the ongoing EU Horizon 2020 funded research projects. Then, we screened the results to identify cases with available data on governance. Cases without information or cases with insufficient information were excluded. Then, we screened the 28 remaining cases in the form of a qualitative content analysis (Mayring 2007) on project documentation, related press releases, project descriptions, case website contents, publications of scientific monitoring and articles available online. If information for few variables could not be found online, we contacted the person in charge for the respective projects for the missing information. For two cases, a full telephone interview was needed to gather the requested information. For ten cases, information could not be collected because either staff turnover did not allow us to contact the person in charge of the project, and the staff was not able to provide the necessary information or we were not able to reach a person in charge of the project by e-mail or phone. 17 cases (Fig. 2 and Table 1) could be fully documented for analysis and interpretation.

**Table 1:** Overview of the selected case sites

Full Title (including location)	Short Title (for figures and tables)	Year	Cost (USD)	Description
Polder Management in Altenheim, Baden-Wuertemberg	Polder Management	1987	773 185	Project improved the riverine ecological functionality and created floodable space dedicated to recreational uses. Implemented measures included floodplain restoration and management, the restoration and reconnection of seasonal streams, the reconnection of oxbows, and the implementation of forest riparian buffers. Challenging issues were forest management, rising groundwater levels and potential increase of mosquito population
Ruhr River Restoration in Binnerfeld, Arnsberg-Neheim, North Rhine-Westphalia	Ruhr Restoration	2006–2011	1 215 005	Project was implemented on a total river length of 4.5 km to stimulate river dynamics, to improve its ecological status and structural diversity as well as flood protection. Measures included the removal of bank fixation to initiate bank-side erosion, the creation of flood-prone areas, the widening of the river bed, the creation of side arms, the restructuring of the river bed and banks by sediment addition and the placement of large pieces of wood
Lahn River Restoration in Cölbe, Hesse	Lahn Restoration	2000	–	Project intended to improve the river hydro-morphological status and functions by removing bank fixation, initiating bank-side erosion, creating side arms and restructuring of river bed and banks
Lech River Restoration in Donauwörth, Augsburg, Bavaria	Lech Restoration	Since 2013	966 481	The project intends to improve the ecological status and functions of the river Lech from the south of Augsburg to the mouth of the river. The regional water management authority in charge uses a Living Lab approach to include various stakeholders and citizens in the development of suitable and widely accepted solutions
‘Living Lab Deusenberg to the Huckarde’ in Dortmund, North Rhine-Westphalia	Emscher Corridor	2018–2023	1 300 055	Besides creating green infrastructure with multiple benefits, one central aspect of this project is also rain water management and reducing rainwater runoff. The project includes community-based urban farms and gardens, food forests, a permaculture orchard and the introduction of pollinator friendly plants using a Living Lab approach and involving citizens for monitoring
Wetland Restoration at Duemmer Lake, Osnabrück, Lower-Saxony	Weser Restoration	2007–2012	3 424 105	The wetlands were restored due to the *European Development Fund* in order to address the Habitats Directive, and Birds Directive. Measures implemented in 2007 and 2012 included the restoration of meadows and pastures, reduced tillage, and a reduced stocking density. A wide variety of stakeholders were involved in the implementation process, ranging from nature conservation agencies and NGO’s, as well as water managers to local farmers
Lippe Floodplain Restoration in Klostermersch Lippstadt, North Rhine-Westphalia	Lippe Restoration	1991–1997	2 209 100	Project intended to stop incision of the river and to improve the ecological status of the river. Starting in 1991, the intensity of the land use was gradually reduced. Fortified embankments were lifted and the river was broadened to 42 m to permit natural dynamics. To stimulate the development of rich structured half-open floodplain landscapes, grazing with Heck cattle was introduced. Restoration measures were discussed with different interest groups and private land owners were invited to participate
Spree River Restoration at Mönchwinkel Grünheide, Brandenburg	Spree Restoration	2013	1 855 644	Project aimed to stabilize the riverbed and improve riverine ecological status. Side arm meanders were reconnected to the river to slow down flows, reduce incision and enhance the ecological qualities of the river. The project was coordinated by the state involving interest groups and NGOs. The renaturation lead to intense controversies, as local land owners and residents were affected by rising water levels and feared damages caused by more frequent flooding events and accumulation of sediment in the river
‘Nature in Grey Zones’ in Duisburg (North Rhine-Westphalia), Erfurt ( Thuringia) and Wiesloch ( Baden-Wuertemberg)	Desealing cities	2013–2016	703 598	Project encourages land owners, e.g., companies and private persons to green up their paved areas in three case study cities to enhance biodiversity and to improve rain water management. Together with a central coordination point and citizen foundations, private companies and land owners as local partners, the three pilot cities of Erfurt, Wiesloch and Duisburg, redesigned paved areas to natural green spaces
Erft River Restoration in Weilerswist, North Rhine-Westphalia	Erft Restoration	2002–2009	791 962	To develop a structure-rich, ecologically permeable stretch of the river with regularly flooded meadows and a high potential for self-development, the dam has been removed and groynes have been built in the river bed to add morphological diversity and initiate lateral erosion
Green Roof Strategy in Hamburg	Green Roofs	2014–2019	3 313 650	In the climate change adaption plan, the green roof strategy is part of the “Urban and landscape planning” action field for climate friendly urban development but also for rain water management. The project subsidizes greening of roofs for at least 70% of both the new buildings and existing suitable roofs
Inner-City-Discharge Program in Hamburg	Runoff Control	2009	-	Two main sewers were rehabilitated to reduce discharge and overflows caused by heavy rain to urban water bodies. The program was part of a project on integrated stormwater management for the city of Hamburg
‘Stream Action Day’ in Hamburg	Stream Restoration	2006	2507	Stream restoration measures were carried out on a few streams such as the Osterbeek (220 m section) and the Middle Bille (150 m section) to implement the Water Framework Directive. The morphology of the river bed and embankments were improved to upgrade the watercourse structure and thus create habitats for typical flora and fauna for this location
Flood Protection and Nature Conservation at Polder Holter-Hammrich, Leer, Lower-Saxony	Green Polders	2008–2011	13 917 330	In order to combine nature conservation and flood protection, various measures were implemented, e.g., dike reinforcement, construction of a new polder canal, conversion to extensive agricultural use and creation of wet shallow water zones
Elbe Dyke Relocation in Lenzen, Brandenburg	Dyke Relocation	2002–2011	14 359 150	In this project, a dike was relocated, reconnecting the river to the floodplains and afforestation of a floodplain was accomplished. The biosphere reserve "Flusslandschaft Elbe-Brandenburg" initiated the project and coordinated stakeholders participation
Cold Air Corridors in Stuttgart, Baden-Wuertemberg	Green Infrastructure	–	-	This project created green infrastructure corridors to reduce runoff, decrease heat waves and to purify urban air. NGOs were involved in planning processes by legal binding consultation procedures
Isar River Restoration in Munich, Bavaria	Isar Restoration	2000–2011	38 659 250	Intensive collaborative planning between numerous stakeholders and a large public participation process lead to new life for the Isar project. It had multiple goals including the improvement of the ecological status of the river, the decrease of the flood risk, and the improvement of the riverscape and recreational potential

### Data analysis and interpretation

The collected project data were transferred into a spreadsheet and prepared for statistical analysis by coding variables to numeric values (Table S1). Variables for which there was no information found were excluded from the analysis. The three variables that were excluded were coordination procedures, exchange platforms to support the participatory process, and participation process intensity and frequency. The codified data set was then assessed by applying exploratory multivariate data analysis using R version 3.6.2 in order to identify patterns and similarities across the cases (*p *< 0.05).

First, an agglomerative bottom-up hierarchical clustering algorithm was used for an initial identification of groups of similar cases. Hierarchical clustering was chosen as it is commonly considered suitable for smaller sample sizes. The dissimilarity matrix for clustering of cases is computed as the Gower distance metric (Gower 1971) which is suitable for mixed-type (categorial and numeric) data (Maechler et al. 2019). The complete linkage criterion was applied.

Then, a Multiple Correspondence Analysis (MCA) was applied to uncover the underlying structure of the data, i.e., the combinations of, and association between factors that govern the dissimilarity of cases in the groups identified and to subsequently describe and refine them. MCA is well suited for the determination of associations between categorial data (Greenacre 2006; Husson et al. 2017). MCA is commonly used for the identification of groups of individuals with similar profiles, e.g., in answer patterns of surveys, as well as to elicit associations between variable categories. The MCA was applied to the full data set. The categorical variables were included as explanatory variables, and numerical variables were included as supplementary information. The first three principal components chosen cumulatively account for about 40% of the variance in the data. The first component alone accounts for about 15% of variance. The second and third dimension, account for about 13.2% and 11.6%, respectively.

Next, in order to refine the initial cluster findings, the cases were subsequently clustered using hierarchical clustering on principal components (HCPC) (Le et al. 2008) using k-means method to allow agglomerative clustering of multivariate data with different metric and structured into themes (Husson et al. 2017). Thus, the most descriptive predictors were identified for each cluster of governance models. Finally, the identified types of governance models were qualitatively compared to existing types of governance models defined in the literature.

## Results

**Fig. 3:**
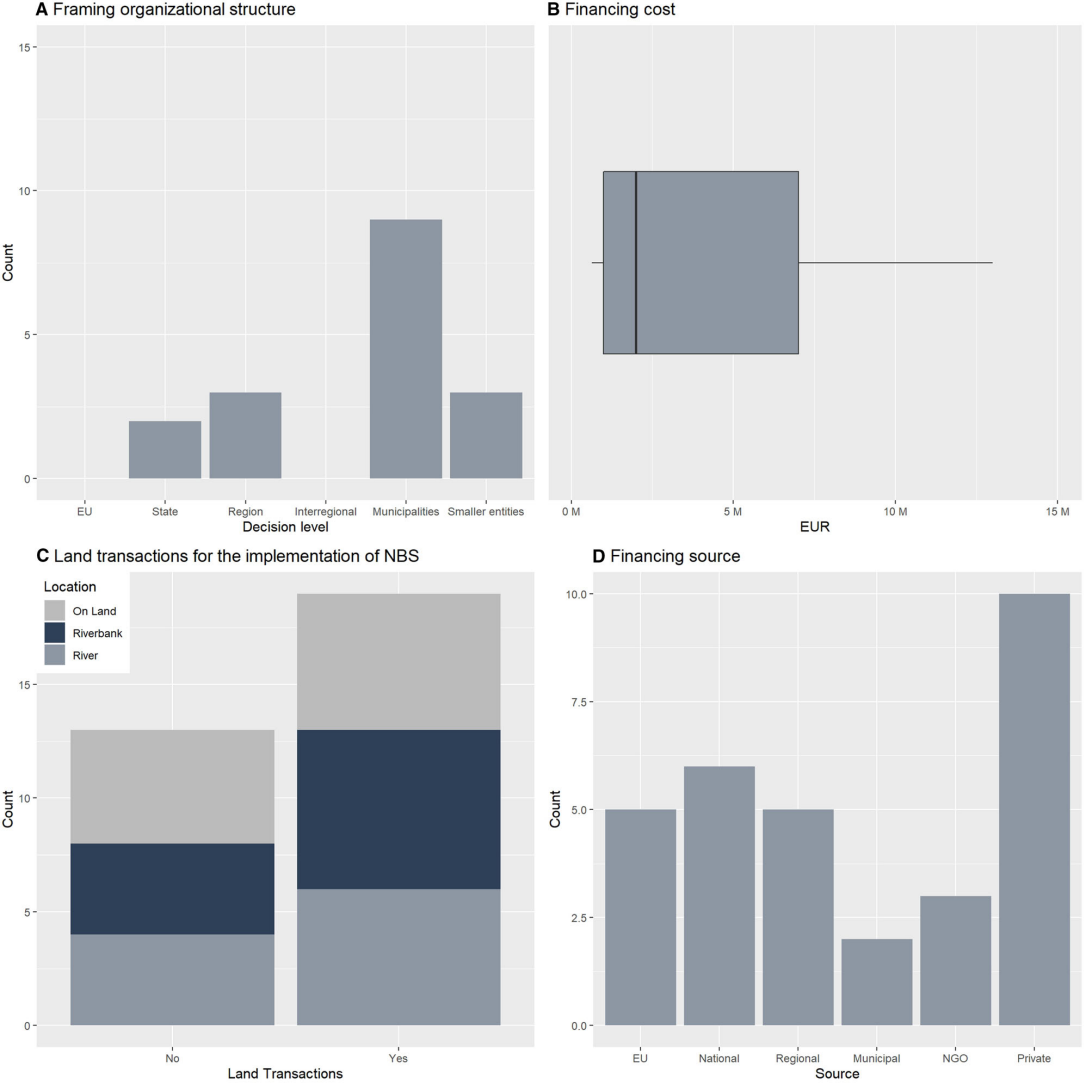
Overview of cases. **a** Case number per decision level as framing organizational structure; **b** box plot of financing costs (excluding outliers); **c** land transactions for the implementation of NBS on land, riverbank, and river locations; **d** number of financing sources

The 17 cases (Fig. 2 and Table 1) showed a broad spectrum of NBS ranging from river restoration to green roofs. Only one of the NBS served a single goal, while the rest had multiple purposes. More than the half of the projects were in the framework of city governments (9 of 17 cases), but most of the NBS resulted from a cross-sectorial decision process (12 of 17 cases). Most of the projects have been implemented under the lead of the city (*N* = 7) or regional (*N* = 6) government. Information on project costs were available for 15 NBS, which totaled to approximately 1.6 million USD (Fig. 3). 70% of the NBS received money from multiple sources (12 of 17), and most of them were funded by public subsidies (e.g., European Agricultural Fund for Rural Development) (15 of 17). 65% of the projects we studied (11 of 17) have been implemented outside of floodplains to reduce runoff, e.g., green roofs. Only 60% of the projects required land acquisition from the private sector. Four projects included measures implemented either in the riverbed and at the riverbank or in the wider surrounding landscape.

**Fig. 4:**
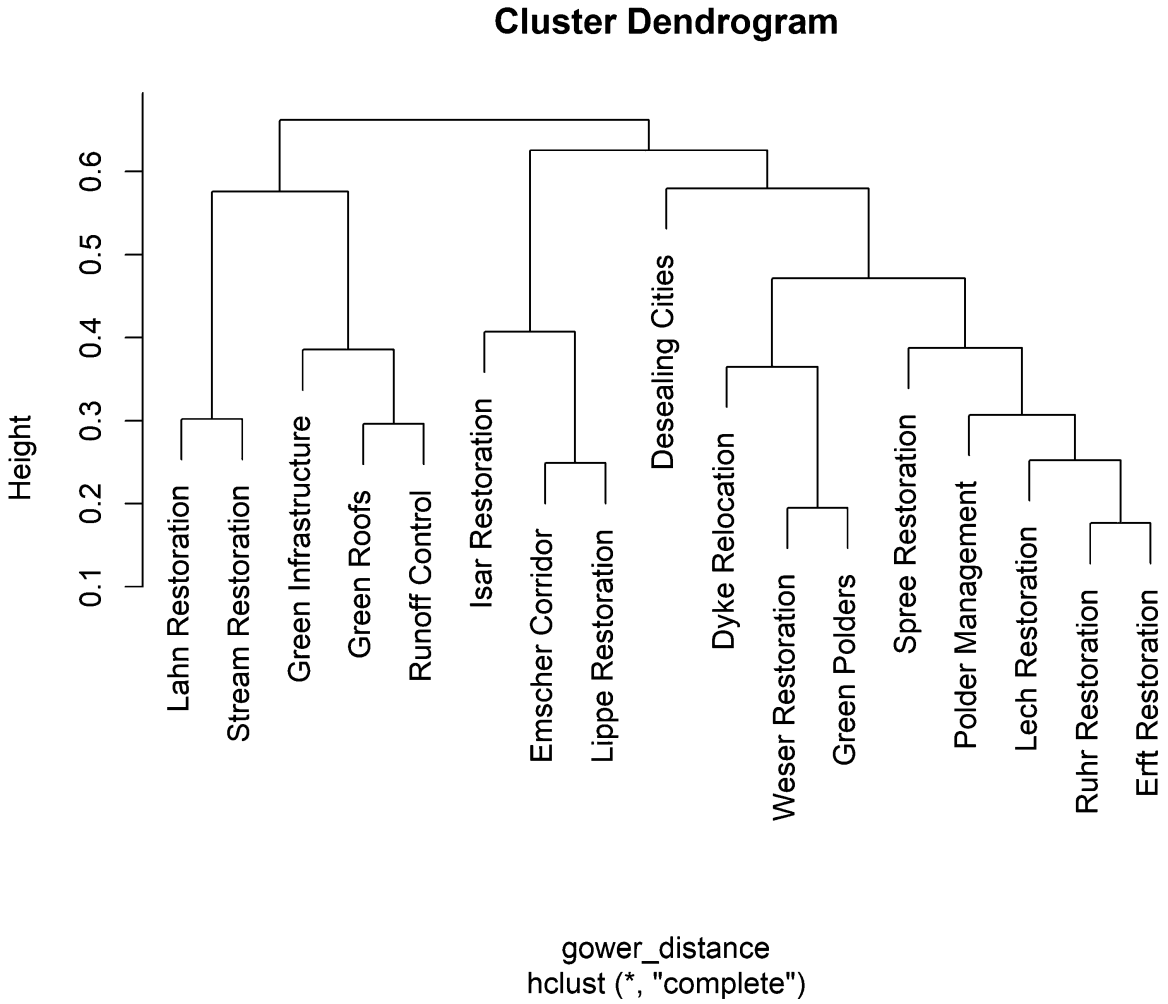
Cluster dendrogram obtained through hierarchical clustering of cases using the Gower distance as a dissimilarity measure and complete linkage criterion to reveal similarity of cases, i.e., common occurrences of predictor factors

The initial assessment of case similarity based on the hierarchical cluster analysis computed from the Gower dissimilarity matrix (Fig. 4) indicated one isolated case (e.g., Desealing cities) and the following similar cases (e.g., Lahn Restoration and stream restoration).

**Fig. 5:**
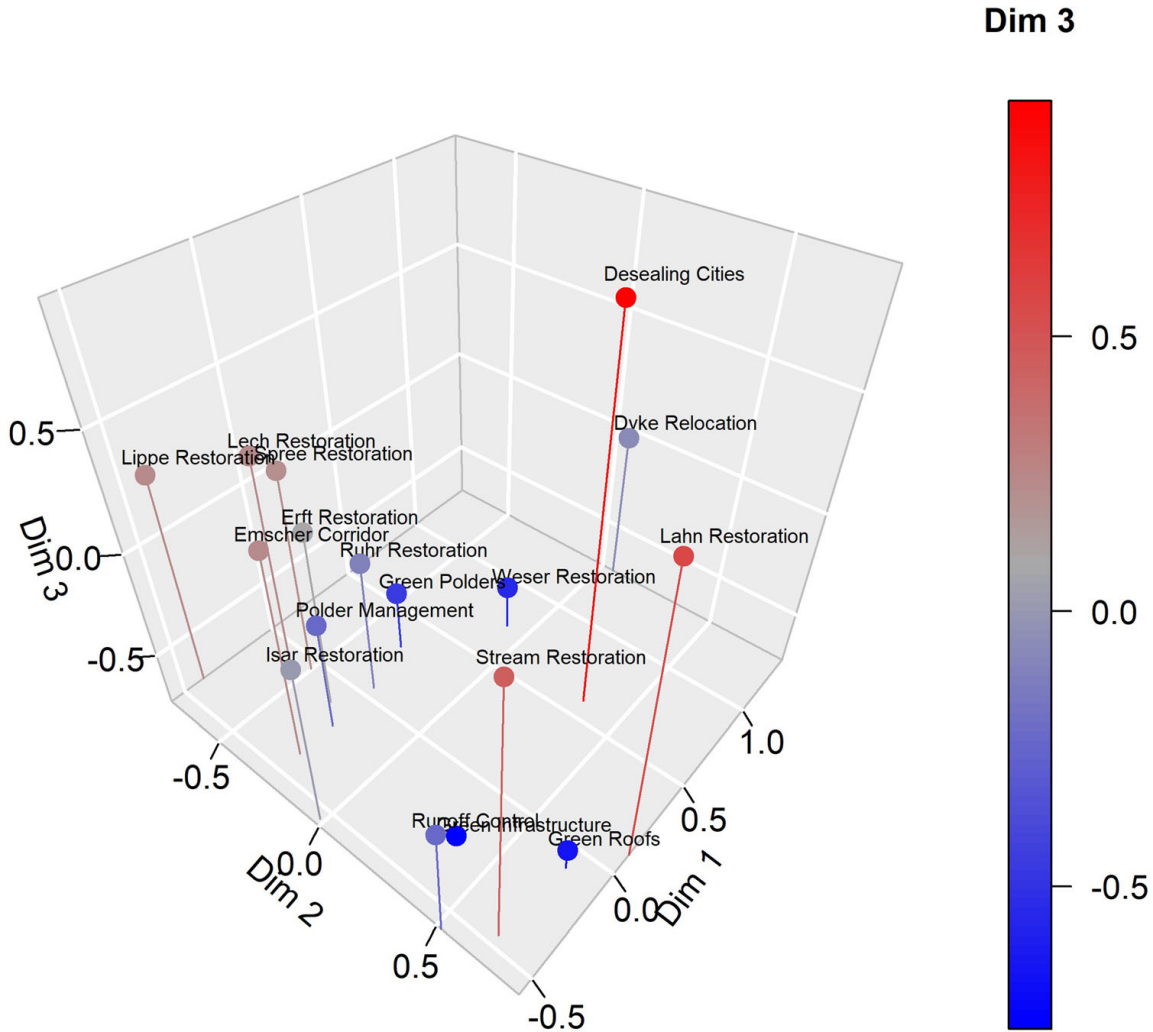
Map of individuals along the first three dimensions. The color of the cases indicates the factor loading on the third component, i.e., blue individuals have negative loads on the third principal component, whereas red individuals load highly on said dimension

The MCA (Fig. 5) distributed the cases in the three-dimensional space that is spanned by the first three principal components (Table 2) and further described by case typology. In particular, MCA results showed that runoff control, green roofs, and green infrastructure form a group that was negatively loaded in the first dimension, positively loaded in the second component, and negatively loaded in the third dimension. Furthermore, the results suggested that stream restoration, Lahn Restoration, and desealing cities were loaded distinctively high in the third dimension, which means that they were characterized by entities smaller than municipalities as the dominant decision level in implementation and participation, and high participation levels such as in co-decision-making and co-design. This exception appears to be in line with the hierarchical cluster analysis that identified the Lahn Restoration, and stream restoration as comparatively similar to each other but rather different to the remaining cases. This is also the case for desealing cities.

**Table 2:** Description of the 5 first principal components of the first three dimensions of the MCA (at p < 0.05)

Variable	Modality	R2	Estimate
1st Dimension			
Institutional setting of the project…	…interplay mechanisms	0.68	0.80
Financing source …	… NGO	0.53	0.56
Implementation at the level of …	… the state	0.67	0.85
Participation in the decision at the level of.	… the state	0.66	0.84
Lead coordinating actor …	… the state	0.63	0.68
Property rights …	… other than state or municipality	0.43	0.34
2nd Dimension			
Financing source …	… regional funds	0.63	0.37
Land transactions…	… are not necessary	0.49	0.33
Decision level of the implementation …	…smaller entity than municipality	0.63	0.58
Participation of the decision at the level of.	… the EU	0.33	0.28
Participation of the decision at the level of.	…smaller entity than municipality	0.52	0.24
3rd Dimension			
Financing source…	…private	0.37	0.35
Decision level of the implementation…	…smaller entity than municipality	0.55	0.60
Participation of the decision at the level of.	…smaller entity than municipality	0.51	0.60
Institutional setting of the project…	… state	0.44	0.89
Participation lead…	…central	0.30	0.26

**Fig. 6:**
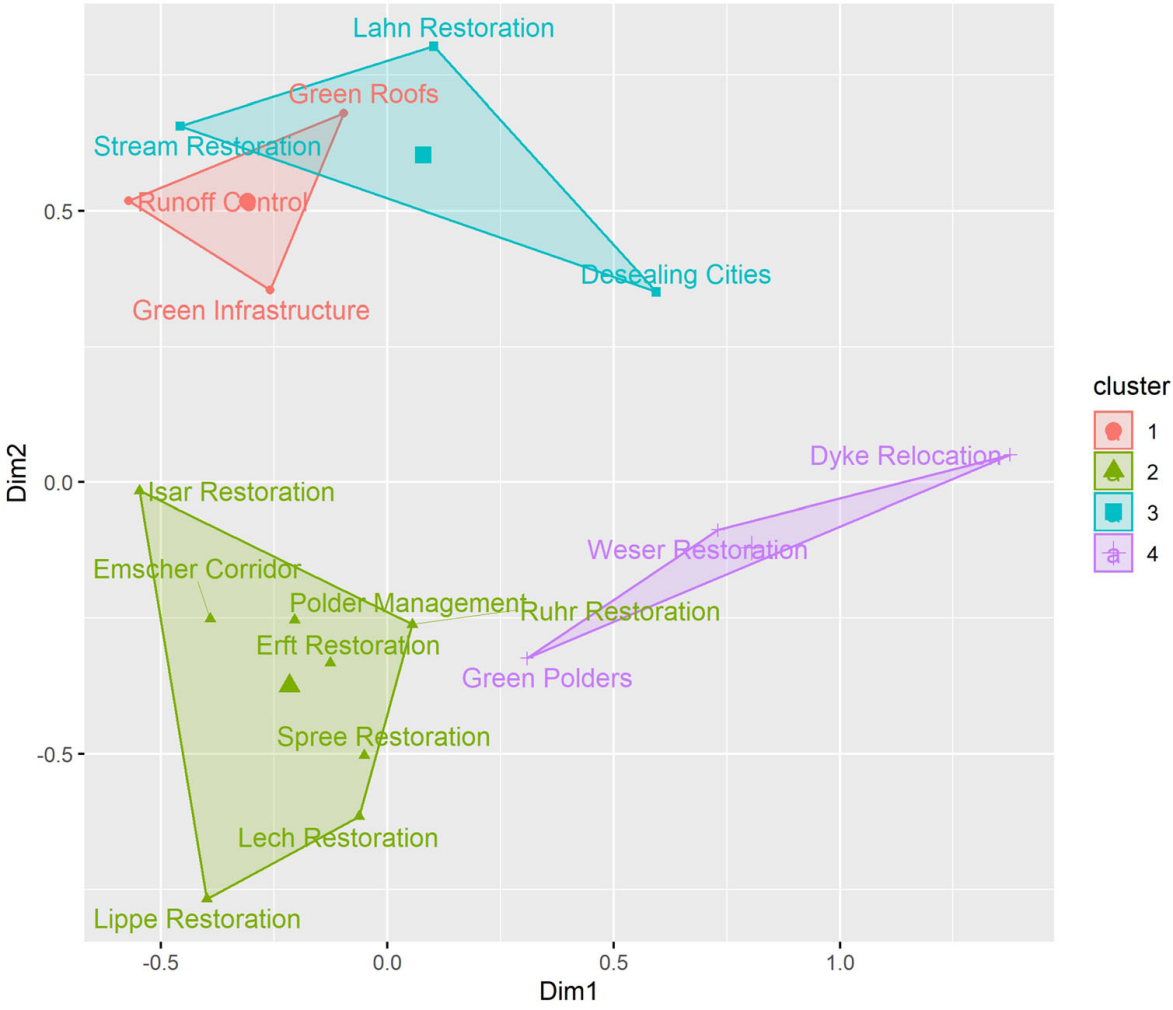
Cluster solution obtained through the hierarchical clustering of principal components, suggesting four clusters as the optimal number of groups

The HCPC cluster algorithm suggests a four-cluster solution (Fig. 6 and Table 3):

**Table 3:** Main project-type characteristics to which project types are associated (at *p* < 0.05)

Governance features	Type 1 Cooperation and Initiatives	Type 2 Co-Design	Type 3 Citizen Power	Type 4 Top-Down
Framing and implementing organisational structures			Entities smaller than the municipalities as the dominant implementation level (100%)	Implementation under the lead of the State (70%)
Project coordination	State			
Participation level	Central	Co-design	Citizen power	
Institutional setting	City government and private		Entities smaller than the municipalities as the dominant decision level (100%)	EU, Decision taken at the level of the State (70%)
Financing model	No regional funding Municipal funding (100%)	Regional funding (100%)	Private contributions (70%) or Municipal	EU and State (100%)
Property rights constellation	No land transactions	State or City government (100%)		
Localization	River bank (100%)	River restoration		No implementation in the river bed

**Fig. 7:**
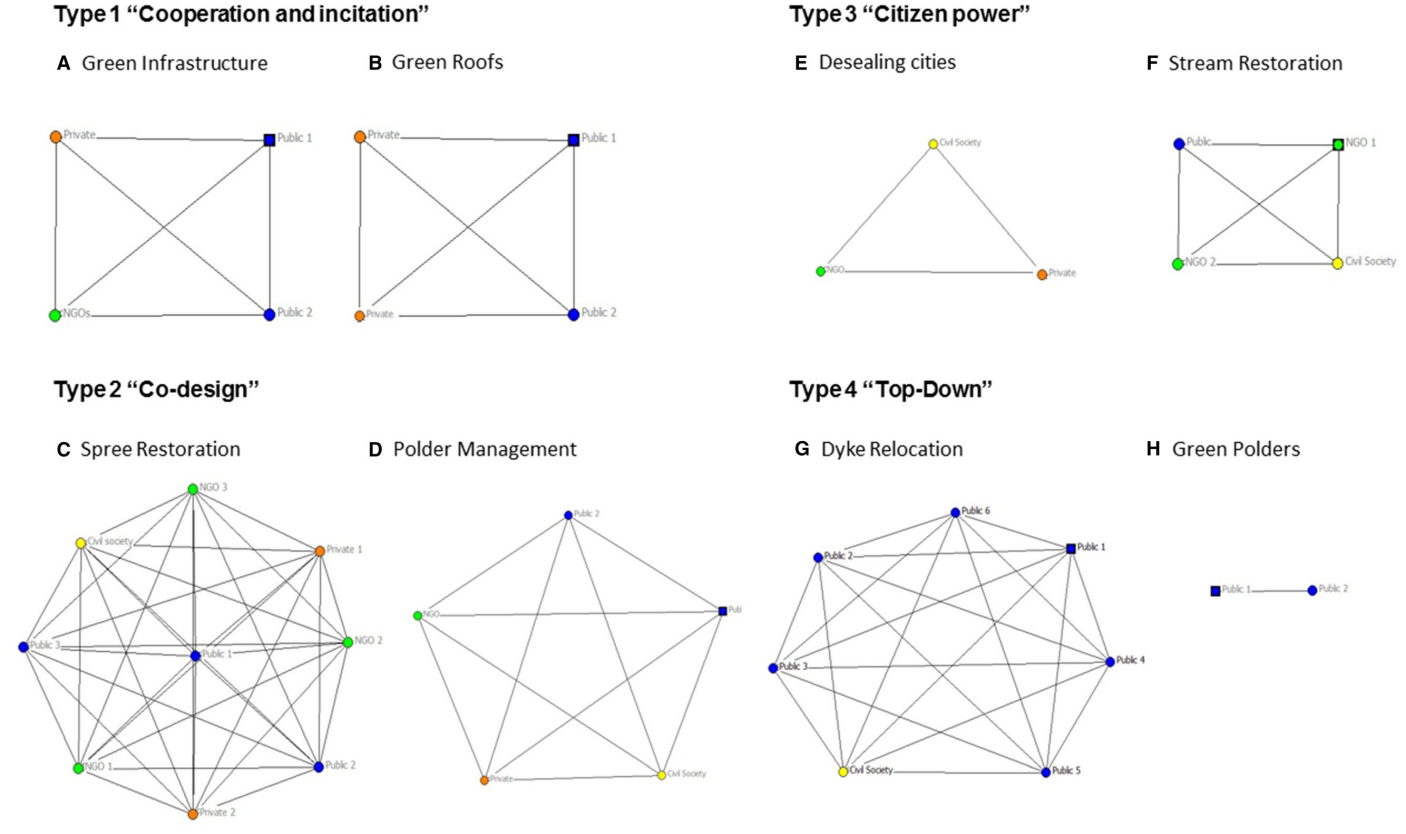
Simple visualization of the network of actors for each case. Each dot correspond to an actor and lines correspond to cooperative exchange to design NBS. Public actors are colored in blue, private actors are colored in orange, NGOs are colored in green, and civil society actors are colored in yellow. The coordinating actor when known is indicated with the symbol of a box

The type 1 cluster (“Cooperation and Incitation”) contains runoff control, green roofs, and e green infrastructure. All of these cases are at least partly funded by the city government, but only half of the cases funded by the city government are in the type 1 cluster. None of the type 1 cases implemented measures at the river bank. The most representative case is green infrastructure, as represented in Fig. 6 by the closeness of this item to the gravity center of the cluster. The stakeholder analysis (Fig. 7) showed the importance of the public–private cooperation to design and implement the NBS. Nevertheless, the planning process still resulted from a central decision center. The mean project cost of the type 1 cluster was 48 600 200 USD.

The type 2 cluster (“Co-design”) is the biggest cluster and is composed of the Spree Restoration, polder management, the Lech Restoration, the Erft Restoration, the Ruhr Restoration, the Isar Restoration, the Lippe Restoration, and the Emscher Corridor. All these projects except the Emscher Corridor implemented measures in the riverbed, and these projects make up 80% of all the river restoration projects. All of the projects benefited from already having some land available for use since the state or municipality that owned the land required for NBS implementation was also the project leader. There were a few instances where some land acquisition was still needed. 87.5% of projects in the type 2 cluster were funded by regional agencies. All the cases that used co-design as participatory forms are type 2 projects. The cluster is best characterized by the Erft Restoration and least characterized by the Lippe Restoration. The stakeholder analysis (Fig. 7) showed that a great diversity of actors from the public, private, civil society, and NGO categories were participating in the design and implementation of the solution. The type 2 cases are the most expensive cases with a mean cost of 187 773 500 USD. This is more than 500 times more expensive than type 3 projects.

The type 3 cluster (“Citizen Power”) is composed of the Lahn Restoration, *The Stream Action Day* in Hamburg, and desealing cities. The case closest to the cluster center is the Lahn Restoration. The characteristics of this cluster are the decision levels of implementation and participation that are composed of entities smaller than municipalities. The funding was mainlyfrom private contributions. No land acquisition is required. The stakeholder analysis (Fig. 7) showed the importance of civil society and NGOs. The type 3 cases are the cheapest with a mean cost of 353 456 USD.

The type 4 cluster (“Top-Down”) is composed of the Weser Restoration, the *Flood Protection and Nature Conservation* at the Holter-Hammrich Polder, and dyke relocation with wetland restoration being the most characteristic case. All of the projects are decided by the EU and funded by both the EU and the state. The implementation requires land acquisition from private owners. The stakeholder analysis (Fig. 7) showed the dominance of the public component. The mean cost of type 4 projects were 10 603 680 USD.

The qualitative comparison between the types of governance models identified and governance model types described in the literature is synthesized in Table 4. On the one hand, there were some similarities between the government model types, but on the other hand, there were also clear differences between the model types based on the observation of practices (method presented in this paper) and model types described at the theoretical level (method found in literature).

**Table 4:** Synthesis of the main results of the governance model typology

Governance models identified	Dimensions	Description	Politics	Reference
Hierarchical governance Network governance Market governance	Formality of institutions and the role of state versus non-state actors	The hierarchical style is characterized by the dominant role of the government, while the network mode includes all forms of cooperation between government and non-state actors. In the market mode, the government delivers services to non-state actors but choices are free and ruled by prices and negotiations	Water	Pahl-Wostl (2015)
hierarchical governance Co-governance Self-governance	Role of governmental and non-governmental actors	Hierarchical governance has at one end of the spectrum a top-down influence by the government and at the other end, self-governance where actors are not controlled by government. Co-governance where public and private actors interact with each other is located in between the two ends	No specific politics	Kooiman (2003)
Hierarchical governance Closed co-governance Open co-governance Self-governance	Actors, power and rules	Amplification of Kooiman´s spectrum by dividing co-governance as closed and open co-governance. Closed co-governance contains a selected mixed group of actors, restricted cooperation and pooled power relations while open co-governance contains a large mix with diffused power and flexible rules of cooperation	Nature policy	Arnouts et al. (2012)
Coercion Voluntarism Targeting Framework regulation	State intervention versus societal autonomy but along the three dimensions of polity (political form), policy (policy content) and politics (political processes)	This typology puts emphasis on the role and the self-empowerment of the state and integrates the European multi-level governance system. Important criteria are, whether legislation is binding; and whether implementation is rigid	European Union	Treib et al. (2007)
Hierarchies, Markets, and Community-based approaches	Power of decision making and resource allocation	Hierarchies are based on command and control and resource allocation occurs through authority and power structures. Market-based approaches are driven by the voluntary exchange among individual actors, and resource allocation is based on willingness to pay. Community management is based on cooperation among actors, and resource allocation is taking into account individual as well as common goals	Ecosystem Services	Vatn (2010)
Centralized governance Decentralized governance Public–Private governance Interactive Governance Self-governance	Actor features, institutional features and feature contents	Models are distinguished according to initiating actors, stakeholder position, policy level and power base (actor features); model of representation, rules of interaction, and mechanism of social interaction (institutional features); and goals and targets, instruments, policy integration, and science-policy interface (features content)	Environmental governance	Driessen et al. (2012)
Self- governing Governing by provision Governing by authority Governing through enabling	Government vs. other actors	Governance modes vary according to the capacity of local government and practice to deliver particular forms of services and resources up to the traditional forms of authoritarian regulation	Climate	Bulkeley and Kern (2006)
Cooperation and Initiatives Co-Design Citizen Power Top-down	Framing and implementing organizational structures Project Coordination Participation level Institutional setting Financing model Property rights, constellation and localization	Governance models range from more participation and private funding to more top-down ruling and state funding	Nature-based solutions	This contribution

## Discussion

This paper has investigated governance models for the implementation of NBS for mitigating flood risk in Germany. The selection of NBS followed a systematic method, and the resulting data set showed a wide variety of implemented measures and few data on governance. Cases in each cluster share distinctive similarities in their governance features. However, the HCPC showed four governance models: (1) “cooperation and incitation”, (2) “co-design”, (3) “citizen power” and (4) “top-down”. This mirrors the whole spectrum of participation level ranging from single information to decision making (Arnstein 1969).

The diversity of stakeholder groups and the direction of mainstreaming at the operational and institutional level differentiate the clusters. Type 1 projects contain NBS such as green roof design with a dominant goal in climate adaptation strategy while also improving flood risk mitigation. These projects intend to reduce hazard exposure more than the vulnerability of the exposed area since they are implemented in the city or in other landscapes rather than along the river. Because of property rights, many private actors are responsible or involved in the implementation of these NBS which are planned by the city government. Therefore, the linking of on-the-ground actors with the city government to implement long-term and large-scale measures aimed at hazard exposure reduction is crucial to ensure implementation. This is often the case when NBS are related to adaptive behavior such as the change in usage of existing open private green space (Wamsler et al. 2017). The opposite model is the type 3 projects. These projects illustrate how citizens can drive action and develop innovative financing models. Type 4 is different from type 1 and 3 because of the simple top-down style stakeholder constellation. This does not always mean that only one powerful entity drives NBS implementation but that other stakeholders are underrepresented. Most cases are type 2 where NBS are co-designed by complex stakeholder constellations. Type 2 projects are very expensive and rely on funding security and land availability from project leaders. These projects are the most comprehensive in applying the four approaches to reduce climate risks: reducing hazard exposure, reducing vulnerability of exposed area, ensuring effective response during risk and ensuring effective recovery (Wamsler et al. 2017).

Our analysis showed that NBS design and implementation resulted mostly from collaborative planning including stakeholders from single or multiple stakeholder groups, i.e., public, private, NGO, and civil society. The numerous NBS goals may be an explanation for the broad spectrum of stakeholders included in the planning and implementation (Zingraff-Hamed et al. 2019). Because of their inherent makeup, NBS can achieve these multiple goals (Raymond et al. 2017; Cohen-Shacham et al. 2019). Governance models with a large spectrum of stakeholders from different geographic and juridical levels are often regarded to be more effective in facing water issues because of their higher resilience and their capacity to deal with complex systems (Lee 2009; Wuijts et al. 2018). This study showed that these governance models are also effective for the implementation of NBS.

In line with previous studies, the 17 German cases studied are advocated at different levels (Wamsler 2015; von Wirth et al. 2019). This observation has been already made for urban parks (Buijs et al. 2019). However, the results of our study show that municipalities, citizens and NGOs, are important pieces of the stakeholder constellation that drives NBS implementation in urban as well as in rural areas. Previous studies concerning ecosystem-based climate change adaptation measures already suggest that in Germany, landscape planning is most advanced in cities in which earlier efforts in environmental planning led to multiple and decentralized decision centers (Wamsler 2015). While collaborative, interdisciplinary, and interdepartmental governance approaches are key for implementing NBS (Kabisch et al. 2016; Frantzeskaki et al. 2019), our study showed that local authorities have a crucial role in integrating NBS into location-based planning strategies. In particular, the “local champion” has a decisive political role in mitigating natural hazards (Martin et al. 2019). A comparison of ecosystem-based adaptation measures for climate change between German and Swedish cases already suggested that in Germany, committed politicians at the municipal level drive the integration of climate change mitigation measures into landscape planning and thus compensating for a lack of clear guidance from the state and regional level (Edelenbos 2005; Wamsler 2015).

In our study, we compared our results to existing governance model typologies. In contrast to these theoretical models deduced from governance theories, our method is inductive meaning that types are based on real-life governance features of various cases. Inductive methods to develop typology are also used in different fields of policy science (e.g., Mattijssen et al. 2018; Celata and Coletti 2019). We found that our typology is in line with existing governance models described in Table 4, which confirms the validity of our results on a broader scale beyond the 17 cases in Germany. Our typology is relatively close to Arnouts et al. (2012) typology, especially because at the difference of for instance Kooiman (2003) that developed three different governance models (hierarchical governance, co-governance and self-governance) along the dimension of the role of governmental and non-governmental actors, Arnouts et al. (2012) divided co-governance as closed and open co-governance. However, differences exist. Other model as for example, Pahl-Wostl’s governance typology (2015) used another approach and differentiates between hierarchical, network and market governance. Compared to this governance typology, there is a difference in presence of the market dimension. Besides the market dimension however, the models are similar in terms of distinguishing between hierarchies and networks. Treib et al. (2007) seems to better address the initiator of the NBS then our typology. Finally, Vatn (2010) is based on the dimensions of the power of decision making and resource allocation and distinguished between hierarchies-based, market-based, and community-based management. Interestingly, when we compared our typology to the governance models of Vatn (2010), we found that the hybrid models with market elements were not very distinctive. This may be due to data collection limitations. Data on resource allocation, financing models, and property rights constellation were partly lacking. Therefore, the topic of market-based approaches has to be explored in future research. Specifically, business models for NBS need to be investigated whether market approaches are suitable governance models for NBS implementation.

Our systematic approach to identify a governance model typology was based on cases in Germany. However, observation of international cases shows the value of investigating a broader scale. Implemented cases included in the ReConect project show that a high exposure to risks, e.g., in the Austrian Alps, is correlated to type 4 governance models. Type 2 governance models have been institutionalized in the Netherlands for centuries but co-design in this case has led to grey infrastructure rather than NBS. Ongoing Phusicos and ReConect cases are located all around the world and intend to incorporate co-design NBS and cover the four identified types of governance models. These insights showed that although NBS can be the result of a traditional state power model, the interest for cooperation-based models and the effectiveness of these models are growing. This highlights the shift from government to governance (Edelenbos 2005).

To face future water governance challenges, the EU is actively encouraging type 2 governance models by funding research and action projects. The EU identified polycentric governance as a driver for successful NBS implementation (EC 2003) and provided clear guidance encouraging collaborative planning through different policies, e.g., the Flood Directive, the Water Framework Directive and the Public Participation Directive. However, some of the collaborative planning processes to co-design NBS struggle to find consensus. Many challenges (Graversgaard et al. 2017) and lessons (Zingraff-Hamed et al. 2019) from polycentric governance and NBS co-design have been identified. Further research may provide valuable insight on the success of the co-design process in different traditional planning contexts. The investigation of adaptability potential and process to more collaborative models of traditional governance constellation is especially relevant for future governance guidance.

While large-scale NBS is crucial for effectiveness, implementation remains at the level of a pilot area or at a local government scale (Hartmann and Spit 2016). However, the Flood and the Water Framework Directives require management plans that exceed the municipal level. In Germany, two historical governance barriers need to be overcome. First, flood protection strategy historically relied on the regional authorities which did not have jurisdiction over the river catchment area or river basin district (Hartmann and Spit 2016; Brödner 2019). Second, stakeholders are accustomed to implementing technical solutions that address local risks (Lünenbürger 2006; Brödner 2019). A paradigm change is urgently needed to implement large-scale solutions as requested by the EU directives.

Our study has few limitations. This investigation did not identify how local authorities integrate NBS into their plans, policies and strategies. Furthermore, we did not identify the best governance model. Moreover, the results depend on the methods applied. It should be noted that we only used NBS documented in online databases and consequently, only successfully implemented solutions. It is probable that not all existing NBS were included. We also relied on the information presented in these databases, which provided a relatively small amount of information on governance issues. Our data analysis applied standard statistical methods that have already been proven effective in identifying project typologies (Zingraff-Hamed et al. 2017a). However, the relatively low number of cases and the large number of variables addressed influenced the outcomes of the analysis. Finally, the study did not try to identify the characteristics of a successful water governance structure.

## Conclusion

This paper investigates governance model that led to 17 NBS implementation in Germany to mitigate flood risk and provides important insights for researchers and practitioners interested in investigating, successfully designing, and implementing NBS. First, this contribution presents a novel attempt in clustering governance models in an inductive manner instead of deductive one. This investigation shows that different models lead to NBS implementation and suggests that no “one-size-fits-all” model can be identified. However, an important commonality between the governance models exists, namely, the inclusion of different stakeholder groups. This suggests that collaborative governance approaches are a key factor for successful implementation of NBS.

Second, this paper suggests that a high degree of cooperation between the stakeholders improves NBS implementation potential. The EU intends to encourage NBS implementation via polycentric governance. However, local, historical, and cultural differences in governance approaches cause difficulties in implementing collaborative planning and context conditions seem to influence the governance models applied. It is extremely important that NBS research projects analyze governance models systematically. Future governance will be challenged to adapt traditional governance models to implement large-scale solutions with higher number of stakeholders.

Finally, this investigation identifies municipalities, citizens, and NGOs as crucial pieces of the stakeholder constellation to NBS design and implementation. Many empirical but few evidence-based work on governance structures for NBS underscore these results. This contribution addresses this gap. Interestingly, while the importance of on-the-ground stakeholders for the design and implementation process of NBS may sound as common sense, in many governance systems, they are not yet recognized. We hope that with the evidence that this contribution provides, planners and managers will be encouraged to take up the ideas of more inclusive governance models in practice.

## Electronic supplementary material

Below is the link to the electronic supplementary material.

Supplementary material 1 (PDF 154 kb)

## Footnotes

1 www.phusicos.eu.

2 www.reconect.eu.

3 https://oppla.eu/case-study-finder.
