# Supplementary material for: Governance models for nature-based solutions: Seventeen cases from Germany
Source: Ambio. 2020 Dec 31;50(8):1610–27. doi: 10.1007/s13280-020-01412-x (PMC8249549; doi:10.1007/s13280-020-01412-x)
Supplement: Supplementary file 1 — Supplementary material 1 (PDF 154 kb) [file 13280_2020_1412_MOESM1_ESM.pdf]

Ambio

Electronic Supplementary Material
This supplementary material has not been peer reviewed.

Title: **Governance models for Nature-based Solutions: cases from Germany**

Aude Zingraff-Hamed, Frank Hüesker, Christian Albert, Mario Brillinger, Joshua Huang, Gerd Lupp, Sebastian Scheuer, Mareen Schlätel, Barbara Schröter

Appendix S1 Survey form

Governance features	variables	Variable description / definition	Variable operationalization	Case Entry	Description	Reference/Source
Basic case description variables	Location, type of NBS, challenges etc.					
Organizational structure<br>I. Decision / planning process	Actor types	List of actors / kind of actors participating in the project	1st step: list of all participating actors<br>2nd step: build groups of actors (“standardisiertes Verfahren”)<br><br><u>Possible groups:</u><br>Water boards<br>State authorities<br>Local authorities<br>Environmental groups<br>Agricultural groups<br>Industrial and commercial actors<br>State enterprise<br>Private actors<br>NGOs<br>Civil society			
	Framing organizational structure: dominant decision level	Beschreibung der Organisationsstruktur im Hinblick auf Entscheidungsbefugnisse und Kompetenzen	1 = EU<br>2 = State<br>3 = Region<br>4 = Intermunicipalities<br>5 = Municipalities<br>6 = smaller entity than municipality			
	Framing organizational structure: centrality		1 = central<br>2 = decentral<br>x = unknown			
	Orientation of the decision level	Are the decisions rather made with regard to sectoral goals or more sector-crossing with regard to the goals of a territory	1 = sectoral<br>2 = territorial (general purpose decision unit)			
Organizational structure<br>II. Implementation process	Actor types	List of actors / kind of actors participating in the project	1st step: list of all participating actors<br>2nd step: build groups of actors (“standardisiertes Verfahren”)<br><br><u>Possible groups:</u><br>Water boards<br>State authorities<br>Local authorities<br>Environmental groups<br>Agricultural groups<br>Industrial and commercial actors<br>State enterprise<br>Private actors<br>NGOs<br>Civil society			
	Framing organizational structure: dominant decision level	Beschreibung der Organisationsstruktur im Hinblick auf Entscheidungsbefugnisse und Kompetenzen	1 = EU<br>2 = State<br>3 = Region<br>4 = Intermunicipalities<br>5 = Municipalities<br>6 = smaller entity than municipality			

Governance features	variables	Variable description / definition	Variable operationalization	Case Entry	Description	Reference/Source
	Framing organizational structure: centrality		1 = central<br>2 = decentral<br>x = unknown			
	Orientation of the decision level	Are the decisions rather made with regard to sectoral goals or more sector-crossing with regard to the goals of a territory	1 = sectoral<br>2 = territorial (general purpose decision unit)			
	Implementation-collaboration	Joint implementation beyond coordination (developing ideas jointly)	A= A responsible authority<br>B= Partnership (2 actors)<br>C= Consortium of different partners (> 2 actors)<br>D= Steering committee<br>E= other<br>F= single actor (not responsible authority)			
Coordination	Coordinator	Who does coordinate? Which actor is the leader?	Name of actor (or refer to kind of actor?)			
	Coordination direction		1 = horizontal<br>2 = vertical			
	Coordination procedures	Procedures for preparing the policy/ project, or, more specifically, the use of knowledge/ data/ evidence within and between organizations	Basic list of options has to be defined; open list which might be extended by the person fulfilling the matrix			
Participation	Participation type		0= Ignorance (no-participation)<br>1= awareness<br>2= provide information<br>3= engage in consultation<br>4= two-way discussion<br>5= co-design<br>6= co-decision-making			
	Kind / type of actors involved		Use same groups as in actor types in the 1st variable group			
	Frequency / intensity of participatory process	How often are the actors invited to participate?	1 = Weekly to monthly<br>2 = Monthly to quarterly<br>4 = quarterly to twice a year<br>5 = once a year<br>6 = less than one a year			
	Kind of platform for exchange / participation	Which tools / platforms are used to organize participation	Online platform<br>Working group<br>….			
Institutional setting	Institutions affecting measure implementation	types of institutions (policies, formal rules) influencing measure implementation	List institutions			
	Institutions affecting other goals (kind)	types of goals (related to policies, formal rules) being affected by the measure	List goals			
	Interplay mechanisms	Institutional mechanisms such as norms, ideas or incentives/costs that are at play, affecting the implementation				
Financing	Project budget / costs	What are the overall costs of the project?	Cost volume			
	Financing sources		1 = Public source – European level<br>2 = Public source – other international level<br>3 = Public source – national level<br>4 = Public source – regional level<br>5 = Public source – municipal level<br>6 = Private source – NGO<br>7 = Private source – private company<br>8 = Private source – insurance<br>9 = Private source - bank			

Governance features	variables	Variable description / definition	Variable operationalization	Case Entry	Description	Reference/Source
			…?<br>Enable multiple choice			
	Number of financing sources	Number of funding sources per project or measure	Number			
	Financing mechanisms	Public subsidies reward land managers for enhancing or protecting ES. They are funded by governments (sometimes with multilateral or donor support)<br><br>User-driven watershed investments channel payments from water users, such as companies or water utilities acting on behalf of customers, to landholders or other parties in exchange for conserving, restoring or creating NBS<br><br>Trading and offsets allow water users to manage their impacts on watersheds by compensating others for offsite activities	1= public subsidies (e.g. European Agricultural Fund for Rural Development)<br>2= User-driven watershed investments (e.g. bilateral agreements, collective action fund/ water fund)<br>3= Trading and offsets			
	Financing institutions	Which kind of institutions do play a role in the financing mechanisms?	1 = Market<br>2 = State<br>3 = Community<br><br>Enable multiple choice			
	Funding mechanism workload	Especially funding schemes may have a high application workload for applicants due to many requirements	1=Easily accessible<br>2=Difficult to access			
	Mechanism funding amount by main funding scheme	funding schemes may require additional funding sources with varying shares for approving funding (e.g. 80 % by public funding scheme and 20 % by applicant or if allowed organized by applicant but from other sources)	Scheme allows 100% funding or<br>Share of own contribution required			
Property rights	Land ownership structure	Ownership structure of the land	1= church<br>2= municipalities/counties<br>3= state/province<br>4= private ownership<br>5= NGOs			
	Land transactions	Number and type of land transactions carried out and point in time of transactions	Number, type (buy out, expropriation, lease agreements), point in time in relation to project progression			
